# Nurses’ perspectives on old age and caring for adults aged 80 years and older: a cross-sectional study in long-term care

**DOI:** 10.1186/s12912-024-02503-w

**Published:** 2024-11-20

**Authors:** Lena Maria Lampersberger, Christa Lohrmann, Franziska Großschädl

**Affiliations:** Institute of Nursing Science, Medical University of Graz, Graz, Austria

**Keywords:** Ageism, Geriatric nursing, Long-term care, Home care services, Residential facilities, Attitude, Aged 80 and above, Nurses

## Abstract

**Background:**

Older care receivers of long-term care at home or in residential care are in sustained and close contact with nurses. Consequently, nurses’ attitudes towards older adults and their care influences the quality of the delivered care. There is a dearth of research on long-term care nurses’ attitudes towards older adults. We aimed to investigate Austrian long-term care (residential care and home care) nurses’ attitudes towards adults 80 + and towards geriatric care, as well as possible influencing factors like personal and professional contact with older adults.

**Methods:**

An online survey using a cross-sectional design was carried out in fall 2023 with a convenience sample of 875 Austrian nurses (qualified nurses, specialised nurses, nurses without diploma). The questionnaire included three scales: (1) The Aging Semantic Differential which measures general attitudes towards older adults, (2) the Perspectives on Caring for Older People Scale, and (3) the Positive/Negative Contact Scales.

**Results:**

Nurses were found to hold neutral to positive attitudes towards adults 80 + and their care. Attitudes towards older adults and their care were positively influenced by positive contact experiences and few negative experiences in their work environment. Nurses in home care had significantly more positive contact with care receivers and held more positive attitudes towards adults 80 + than residential care nurses.

**Conclusion:**

These results suggest that intergenerational contact can positively impact attitudes of nurses towards older care receivers. In order to further facilitate positive attitudes, it is recommended to create positive contact opportunities between nurses and care receivers by, for example, implementing intergenerational educational interventions.

**Supplementary Information:**

The online version contains supplementary material available at 10.1186/s12912-024-02503-w.

## Background

Due to advances in medicine, education, living conditions, and access to health care, we are now looking forward to living longer. Due to this longer life expectancy, the global population is becoming steadily older [1, 2]. As our population ages, we will face challenges such as how to provide a sufficient measure of health care, but we will also experience possible advantages: Older adults can contribute greatly to our society as active members in the working life, in volunteering, and in their families [2].

Throughout the course of life, diseases and a need for care can and will occur sooner or later [2]. By the age of 80, the risk of care dependency is considerably increased, and approximately two-thirds of older adults are in need of care and support to perform activities of daily living [3, 4]. One of the challenges presented by an ageing society is the necessity to develop and maintain a sustainable care system which can deliver adequate and professional best-practice care to all older adults [2]. This quality older adult care should be governed by a holistic approach to meet all health needs of the older adult population. This objective could be achieved by an integrated care system in which all services providing care and support work together in a coordinated manner to meet the needs of older adults [5].

The occurrence of care dependency, multimorbidity, or frailty marks the beginning of the so-called fourth age of a person, which is not defined by chronological age but by the state of a person’s health and functionality [6]. Older adults, who previously enjoyed health and independence, transition slowly from the third into the fourth age and, consequently, develop a greater need for care and medical services [7, 8]. This transition is mostly presumed to take place between 80 and 85 years of age. When the need for care and help increases, measures such as providing integrated care at home or in a residential facility need to be taken to help older adults maintain their functional abilities, thus increasing the need for long-term care [5]. Long-term care is defined as care and support which enables an individual to maintain functional abilities and *„to ensure that people with or at risk of a significant ongoing loss of intrinsic capacity can maintain a level of functional ability consistent with their basic rights, fundamental freedoms and human dignity.*” ([5], p. 6–7). It is characterised by a continuous or intermittent system of care provided over sustained periods of time and which can be delivered by relatives or friends, professional caregivers, community-based services, or institutional caregivers [5]. Long-term care, therefore, includes both residential care and home care. Older adults aged 75 years and older represent the largest group of care receivers in long-term care [9], with adults aged 80 years and older constituting the group with the highest and most complex care demands [10].

When these individuals are being cared for either at home or in residential care facilities, it is nurses who are in close and sustained contact with people in the fourth age. Tensions and problems may arise between nurses and these older people, leading to ageism towards the older care receivers. In health care and nursing care, ageism is a pressing issue [11, 12]. *“Ageism in the nursing care of older adults is any kind of stereotype, prejudice, or discrimination against or to the benefit of older adult patients that is implicitly or explicitly practiced by the nurse and leads to actual or perceived (direct or indirect) decrease in the quality of health care provided*” ([13], p. 10). Due to the fact that nurses frequently interact with ill and care-dependent older adults, they may form biased opinions towards them during the course of their careers, i.e., negative or ageist attitudes [14–16]. Therefore, the risk of nurses expressing negative attitudes towards adults aged 80 years and older or the latter experiencing ageism increases because they are stereotyped as being frail and dependent [11, 17]. Studies show that frequent and negatively perceived contact with older adults, and especially older care receivers, may affect nurses’ attitudes towards older adults and lead them to display subtle forms of ageism [18, 19]. This influence can be explained by Allport’s contact hypothesis [20].

In the 1950s, Allport’s contact hypothesis laid the foundation for research on the reduction of prejudice by promoting interpersonal contact, albeit originally between ethnic groups. Since then, the contact hypothesis has been used to study different forms of prejudice [21, 22]. In ageism research, Allport’s contact hypothesis is one of several (e.g. social identity theory [23]) commonly used to explain psychological mechanisms that lead to ageism [24]. As close contact between nurses and older care receivers may influence whether older adults experience ageism, this hypothesis is also used to study ageism in the context of nursing [14, 18]. The contact hypothesis suggests that interpersonal contact may diminish prejudice. More specifically, prejudice between two groups may be reduced by encouraging social contact experiences taking place under optimal conditions, while also emphasising the equal status of the respective groups, common goals, intergroup cooperation, and the support of authorities, law, or customs. As the attitudes towards a specific individual become more favourable, the attitudes towards the whole group (e.g. age group) become more positive as well [20]. Although Allport [20] stressed that deeper engagement is needed to reduce prejudice, he also said “the more contact, the more trouble” ([20], p. 263). This means that the contact needs to occur under optimal and favourable conditions, or else it may be experienced as unfavourable, thus leading to more prejudice [20]. Nurses, on the other hand, may not experience contact with older adults under such optimal and favourable conditions, as they usually interact with older adults with a high care demand. Even though Allport’s contact hypothesis suggests a positive effect of intergenerational contact regarding positive attitudes towards older adults, this might not apply to nurses, as their contact experiences often take place under challenging conditions [15]. Especially in long-term care, nurses are in close contact with older care receivers who constitute the largest group of care receivers in this settings and tend to have the most complex care needs [9, 10]. Furthermore, nurses’ personal contact with older adults might also be perceived as positive or negative. Therefore, both professional and personal relationships with older adults could be confounders with regard to nurses’ attitudes towards older adults, especially in view of the nature of the conditions under which this contact takes place [19].

In line with Allport’s contact hypothesis, Drury et al. [18] adapted a scale to measure the quality of nurses’ contact with older care receivers (Positive and Negative Contact Scales, PNCS). We hypothesised that the quality of contact with older care receivers influences nurses’ attitudes towards older adults and their perceptions of geriatric care. Some studies identified contact as a factor that impacts attitudes towards older adults, but no comprehensive body of research exists with regard to this topic. The available results are inconclusive, and there is still a paucity of research, especially with regard to long-term care [14, 18, 25, 26].

Nurses’ attitudes towards older adults affect the quality of care. For example, a nurse’s negative attitudes towards care receivers may reduce the amount of care provided [15, 27]. If, on the other hand, they hold positive attitudes towards care receivers, a better relationship, characterised by trust and comfort, exists between the nurse and the person in need of care [28]. So far, there is only a small pool of literature on nurses’ attitudes towards older adults and their care in the long-term care setting, and especially towards adults aged 80 years and older, and little research has been carried out in recent years in either residential care or home care settings [29]. As care receivers are in close contact with nurses, such research is vital to ensure professional and high-quality care because it provides valuable insight into nurses’ attitudes towards these adults [15, 27, 30]. A systematic review by Rush et al. [15] revealed conflicting results on whether nurses hold positive or negative attitudes with regard to caring for older people. To our best knowledge, no study has examined potential differences in nurses’ attitudes in various long-term care settings (i.e. residential care and home care).

To ensure that older adults age in a healthy way and receive best-practice integrated care, nurses need to hold positive attitudes towards them [2], but no comprehensive body of research exists on nurses’ attitudes towards older adults, their care in long-term care settings, or the influence of the quality of contact on nurses’ attitudes. Therefore, we set three aims in our study:

1. To assess the attitudes of Austrian nurses working in long-term care towards adults aged 80 years and older and towards geriatric care;
2. to compare nurses’ attitudes towards adults aged 80 years and older in the residential care and home care settings; and
3. to assess which factors influence nurses’ attitudes, especially with regard to the role of the quality of contact with care receivers aged 80 years and older.

## Methods

### Design

An online survey with a cross-sectional design was used to describe the relationship between variables [31]. An online questionnaire developed using LimeSurvey 6.5 was sent to nurses working in Austrian long-term care facilities [32]. The Strengthening the Reporting of Observational Studies in Epidemiology (STROBE) checklist for cross-sectional studies guided the reporting used in this study [33].

### Setting and participants

Austrian nursing staff including nurses (with a diploma, bachelor’s, or master’s degree), specialised nurses, and nurses without a diploma working in long-term care were included in this study. These nursing professions were chosen because they provide direct care to older adults in need of care and, thus, were in close contact with these individuals. In Austria, nurses attend a three-year training programme and graduate with either a bachelor’s degree or a diploma. After graduation, nurses can specialise in fields like intensive care or palliative care by completing a one-year university course. Nurses without a diploma are comparable to assistant nurses and nursing aides; they receive one to two years of education. To be included in the study, any amount of time working with older adults in long-term care was sufficient. The long-term care setting included all nurses working in residential care or home care as well as community nurses.

#### Calculation of sample size

In the initial stage of the planning process, the representative sample sizes needed for the residential care and home care settings were calculated separately using Qualtrics’ sample size calculator [34] based on the population size reported by the Austrian government [35]. For the residential care setting, *n* = 381 participants were needed, while *n* = 377 participants were needed for the home care setting.

#### Sampling

A convenience sampling strategy was used. Based on e-mail address lists from all federal states in Austria, including those for residential care facilities, home care services, and community nurses, e-mails were sent to either nursing managers or an available contact e-mail address. The e-mails included a description of the study and the request to forward the link to the study to their nursing staff. A letter was attached to this e-mail which included the study description, an invitation to participate, and a QR code for access to the online survey. This survey could also be printed and pinned up at the work place. A reminder was sent to the same e-mail addresses after one month of data collection. Data were collected using LimeSurvey 6.5 [32] in October and November 2023.

### Instrument

The questionnaire took about ten minutes to complete and had been designed in the course of a previous study [19]. It included demographic questions, the German version of the Aging Semantic Differential scale (ASD) [36, 37] (Cronbach’s α > 0.8 [36]), the German version of the short version of the Perspectives on Caring for Older People scale (PCOP) [19, 38] (Cronbach’s α for the English version < 0.8 [38]), as well as possible influencing factors extracted from the literature (e.g. sex, work experience in long-term care facilities, geriatric/gerontological education, contact with older care receivers) [18, 39–41]. The ASD produces sum scores ranging from 26 to 182, with lower scores reflecting more positive attitudes. Each item has a maximum score of seven, with a score of one indicating a positive, four a neutral, and seven a negative attitude, respectively [36, 37]. The PCOP produces sum scores ranging from 9 to 36, with higher scores indicating a more positive view towards caring for older adults. Items are rated on a four-point Likert scale (1 = strongly agree; 4 = strongly disagree). It consists of 9 items which describe attitudes about the care of older people in need of care (e.g., ‘Caring for older patients is usually challenging and rewarding’, ‘I would not choose to attend continuing education in nursing care of older patients’) [38]. A detailed description of the questionnaire can be found in Lampersberger et al. [19]. For the purpose of this study, the Positive and Negative Contact Scales (PNCS) [18] were also included to assess the quality of contact between nurses and care receivers as a possible influencing factor.

The PNCS consist of two scales: the PCS (Positive Contact Scale) and the NCS (Negative Contact Scale). These are used to measure the quality and frequency of positive or negative contact in the care context. The PCS consist of eight items, three measuring the quality of positive contact and five measuring the frequency of positive contact. The NCS consist of six items, three each measuring the quality and frequency of negative contact. The items are measured on a seven-point Likert scale, with the quality of positive or negative contact ranging from 1 = none to 7 = all, while the frequency of positive or negative contact ranges from 1 = never to 7 = very often. A number ranging between 1 and 49 is obtained by multiplying the means of the quality and frequency of contact in each scale. Means are multiplied due to the differences in the number of items. Higher PCS scores indicate contact with older care receivers that is of good quality and frequently positive. Higher NCS scores indicate contact with older care receivers that is of poor quality and frequently negative. Cronbach’s *α* was calculated as > 0.79 for each part of the PCS and NCS [18]. The authors gave their permission to translate and use the scale.

#### Cross-cultural adaptation of the PNCS

The PNCS was cross-culturally adapted according to Beaton et al. [42] in four phases.

- Phase 1: The scales were forward translated by two independent translators, both of whom speak German as their first language and one of whom was aware of the concept being measured and has a background in nursing science. The translations were then synthesised by one author and sent to the translators for confirmation.
- Phase 2: The translation was sent to two translators whose first language is English for backward translation. Both were not aware of the measurement and the concepts to be measured and had no background in nursing science. The translations were synthesised by one author.
- Phase 3: The translations were discussed in an expert committee composed of the translators and one author of this study and therefore consisted of language professionals, nurses and nursing scientists. After a consensus was reached, the pre-final version of the scales was sent to the author of the scale to receive feedback. No changes were made.
- Phase 4: To assess the clarity and face validity of the scales, a pre-test was carried out with 17 master’s students in nursing science who also were working as nurses. The sample for the pre-test was a convenience sample. E-mails with the invitation to participate were sent to the students. Two examples of pre-test questions are: ‘Please assess whether the following description and items on the Likert scale are understandable’ and ‘If the labelling or items were unclear, what was not clear to you?’. No changes were made.

### Internal reliability of the PNCS and PCOP

The PCNS consists of the scales PCS Quality (Cronbach’s *α* = *0.8)*, PCS Frequency (Cronbach’s *α* = *0.9)*, NCS Quality (Cronbach’s *α* = *0.8)*, and NCS Frequency (Cronbach’s *α* = *0.8)*. All of these scales had good internal reliability with a Cronbach’s *α* ≥ 0.8. Both PCS scales combined, as well as both NCS scales combined, had a Cronbach’s *α* ≥ 0.8 as well. The PCOP had an acceptable Cronbach’s *α* = 0.7 [43].

### Ethical considerations

Ethical approval was obtained from the Medical University of Graz (EK Number 31–320 ex 18/19). When starting the online survey, participants had to read the description of the study and confirm having read the data privacy policy. In response to the first question, nurses had to answer if they were willing to participate in this study. By answering ‘yes’, nurses gave their informed consent. No conclusions could be drawn about the participants’ identities, as the data were collected anonymously. No IP addresses of participants were saved.

### Data analysis

The statistical software IBM SPSS Statistics (Version 28.0) was used for data analysis [44]. First, all questionnaires from participants who did not provide care for older care receivers were excluded. Then, questionnaires with more than 40% missing values were removed from the data pool [45], resulting in *N* = 875 included questionnaires (residential care *n* = 429 and home care *n* = 446). No imputation method for missing values was used in these questionnaires. Because of the number of participants, a normal disruption was assumed [43]. To test the scales for internal reliability, Cronbach’s *α* was calculated for each scale for each setting and for long-term care in total by using the entire sample. Descriptive statistical analyses were performed with respect to the characteristics of the study participants and the scores on the various scales. Means and standard deviation were used for continuous variables, while percentages were used for categorical variables. To identify differences between data from residential care and home care settings, unpaired *t*-tests were carried out for continuous variables. Depending on the variance, Levene’s test or Welch’s *F* test was applied, and two-tailed *p*-values were used. The linear relationship between the ASD sum score and the PCOP sum score was calculated using a scatterplot (jittered) and the Pearson correlation.

To test for influencing factors, two multiple linear regression models were applied. The first model included the PCOP sum score as a dependent variable, whereas the second model included the ASD sum score as a dependent variable. No questionnaires with missing values were included in the multiple linear regression models. All questionnaires from participants stating ‘other’ in the variable *sex* were excluded due to missing values in the questionnaire; thus, a binary variable for sex was used (female/male). For non-binary categorical variables, dummy variables were built. First, all possible influencing variables were included in the models (see Table 1). Then, backward elimination of non-significant variables and theoretical assumptions was applied to exclude variables from the model. Consequently, variables measuring the contact of nurses with older adults and the variable ‘years working in long-term care’ were included in every model (see Table 1) to control for possible confounders. Variance inflation factors (*VIF*) were checked to determine whether they were below 10 to rule out multicollinearity [43]. The assumptions of linearity, independence of errors, homoscedasticity, unusual points, and normality of residuals were met.* P*-values ≤ 0.05 were considered as statistically significant.

**Table 1:** List of predictor variables included in the multiple linear regression models in the beginning

**Variable**	**Category (1)**	**Reference category (0)**
Sex	Female	Male
Age	Continuous variable	
Origin	Non-Austrian	Austrian
Educational level	Academic	Non-academic
Profession^a^	Nursing staff without diploma Specialised Nurse	Qualified nurse
Years working in long-term care^b^	Continuous variable	
Setting	Home care	Residential care
Clinical focus	No	Yes
Geriatric or gerontological education	No	Yes
PCS multiplied score	Continuous variable	
NCS multiplied score	Continuous variable	
Interaction with person in need of care ≥ 80^b^	Often	Occasionally
Able to discuss personal topics^b^	Few/no	Many/all
Thinking of a gender while answering the ASD^a^	Female Male Other	No
Knowing the meaning of ageism^a^	Unsure No	Yes

*PCS* Positive Contact Scale, *NCS* Negative Contact Scale, *ASD* Aging Semantic Differential scale, *PCOP* Perspectives on Caring for Older People scale

^a^Dummy variable

^b^Variables were included in both final models due to theoretical considerations

## Results

### Participant characteristics

In long-term care, nurses had a mean age of 44.8 (± 9.9) years, an average of 18.3 years of experience working in health care in general, and 13.9 (± 8.9) years of experience working in long-term care. Nurses working in home care had a mean age of 45.4 (± 9.1) years and were statistically significantly (*p* = 0.045) older than nurses working in residential care, who had a mean age of 44.1 years. Nurses working in residential care, however, had with an average of 13.9 (± 8.9) years of experience working in long-term care a statistically significantly (*p* ≤ 0.001) greater amount of experience than home care nurses, who had on average 11.8 (± 9) years of experience.

In both settings, most of the participants were female (> 80%) and qualified nurses (51.7%) who interacted daily with care receivers aged 80 years and older (> 75%). Information about participant characteristics, working environments, and frequency of contact with care receivers aged 80 years and older for both settings, as well as statistically significant differences between residential care and home care nurses can be found in Table 2.

**Table 2:** Participants’ characteristics, working environment, and contact with care receivers ≥ 80 years of age in residential care, home care, and long-term care in total

Variable	Category	Residential care		Home care			Long-term care total	
		*n*	*%*	*n*	*%*	*p*	*N*	*%*
Sex		423		435		**≤ 0.001**	858	
	Female		84.4		82.0			88.2
	Male		15.6		7.8			11.7
	Other		0.0		0.02			0.1
Origin	Austrian	429	80.9	445	93	**≤ 0.001**	874	87.1
Education		428		445		**≤ 0.001**	873	
	Non-academic		80.1		89.2			84.8
	Academic		19.9		10.8			15.2
Profession		427		444		0.511	871	
	Qualified nurse		49.9		53.4			51.7
	Specialised nurse		11.5		36.9			10.5
	Nursing staff without diploma		38.6		9.7			37.8
Clinical focus		429		445		**≤ 0.001**	874	
	Geriatrics		60.1		38.7			49.2
	Palliative care		11.9		3.1			7.4
	Psychiatry		1.9		1.1			1.5
	No focus		21		48.3			34.9
	Other		5.1		8.8			7
Geriatric/Gerontological education		429		444		**≤ 0.001**	873	
	No		48		39.6			54.3
	Yes		52		60.4			45.7
	Part of nursing programme^a^		38.1		42			39.8
	Stand-alone course^a^		81.6		82.4			82
	Postgraduate training^a^		12.6		6.3			9.8
	Others^a^		2.2		2.8			2.5
Interaction with persons in need of care ≥ 80		429		446		**≤ 0.001**	875	
	Daily		85.8		76			80 8
	Occasionally		14.2		24			19.2
Having family/friends ≥ 80		429		446		**0.007**	874	
	No		26.6		21.5			24
	Yes		73.4		78.5			76
	Can discuss many or all personal topics		64.9		56.6			60.5
	Can discuss few or no personal topics		35.1		43.4			39.5
Thinking of a gender while answering the ASD		427		443		0.490	870	
	No		80.8		81.3			81
	Female		14.8		16			15.3
	Male		2.1		1.6			1.8
	Other		2.3		1.1			1.7
Knowing the meaning of ageism		402		434		0.369	836	
	Yes		41.8		37.8			39.7
	No		35.6		36.2			35.9
	Unsure		22.6		26			24.4

*ASD* Aging Semantic Differential scale

*p*- values ≤ 0.05 in bold

^a^multiple responses possible

### Nurses’ attitudes towards adults aged 80 years and older and their care

The PNCS showed that nurses had a high quality and frequency of positive contact with care receivers. Participants experienced negative contact to a low extent and frequency. Nurses held neutral to positive attitudes towards adults aged 80 years and older and perceived their care positively. Home care nurses had statistically significantly more positive contact with older adults and more positive attitudes towards them. Table 3 shows the mean scores on the PNCS, ASD, and PCOP scales as well as differences between settings. Supplementary File 1 shows the radar charts per scale and setting including the means per item. These results show that participants working in home care reported more positive and less negative contact with care receivers aged 80 years and older. No adjective pair on the ASD was rated negatively, and no difference between settings was identified regarding the PCOP.

**Table 3:** Participants’ quality of contact with care receivers, their attitudes towards older adults and geriatric care in residential care, home care, and long-term care in total

Scale	Residential care		Home care			Long-term care total	
	*n*	Mean score (SD)	*n*	Mean score (SD)	*p*	N	Mean score (SD)
PCS Quality	429	5.3 (0.9)	446	5.6 (0.8)	**≤ 0.001**	875	5.4 (0.9)
PCS Frequency	429	5.1 (1.2)	446	5.4 (1)	**≤ 0.001**	875	5.3 (1.1)
NCS Quality	429	2.6 (1.1)	446	2.3 (1)	**≤ 0.001**	875	2.4 (1)
NCS Frequency	428	2.4 (0.9)	446	2 (0.7)	**≤ 0.001**	874	2.2 (0.9)
PCS	429	27.7 (9.5)	446	30.7 (8.6)	**≤ 0.001**	875	29.2 (9.2)
NCS	428	6.9 (5)	446	4.8 (4)	**≤ 0.001**	874	5.9 (4.6)
ASD	428	3.7 (0.9)	443	3.6 (0.8)	**0.01**	871	3.7 (0.8)
ASD – Instrumentality	428	4 (0.9)	445	3.9 (0.9)	0.146	873	3.9 (0.9)
ASD – Autonomy	428	4 (1.1)	446	3.9 (0.9)	**0.025**	874	4.0 (1)
ASD—Acceptability	428	3.3 (1)	444	3.0 (0.9)	**≤ 0.001**	872	3.2 (1.0)
ASD—Integrity	428	3.7 (1.1)	445	3.7 (1.0)	0.371	873	3.7 (1.0)
PCOP	408	3.2 (0.4)	435	3.3 (0.4)	0.224	843	3.2 (0.4)

*PCS* Positive Contact Scale (mean score for quality and frequency ranging from 1 to 7, higher scores indicating more positive contact, mean total sum score of the PCS scales ranging from 1 to 48, higher scores indicating more positive contact), *NCS* Negative Contact Scale (mean score ranging from 1 to 7, lower scores indicating more negative contact, mean total sum score of the NCS scales ranging from 1 to 48, lower scores indicating more negative contact), *ASD* Aging Semantic Differential scale (mean score ranging from 1 to 7, lower scores indicating more positive attitudes towards older adults), *PCOP* Perspectives on Caring for Older People scale (mean score ranging from 1 to 4, higher scores indicating more positive attitudes towards geriatric care), *p*- values ≤ 0.05 in bold

### Correlation between the PCOP and the ASD

**Fig. 1:**
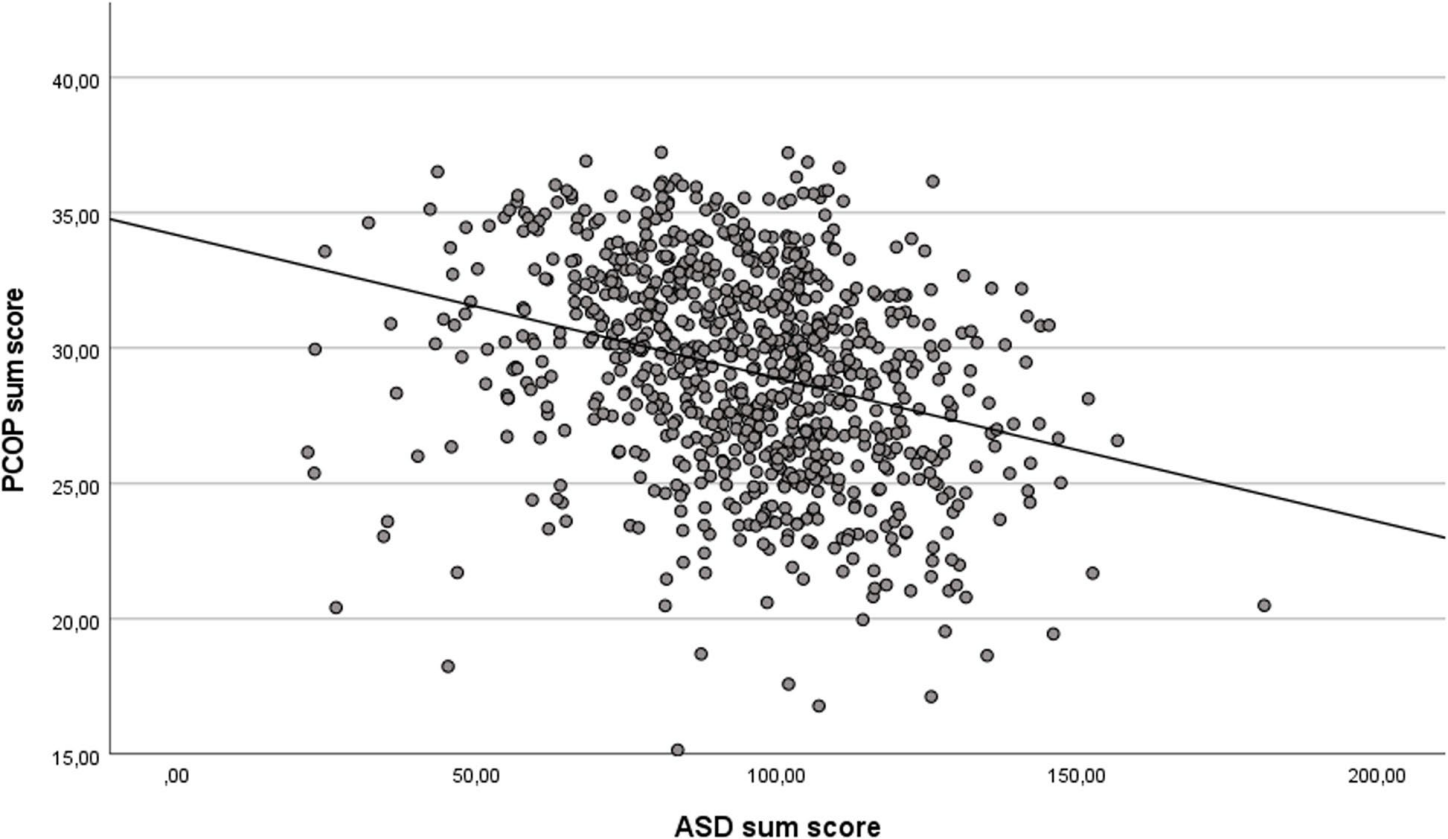
Scatterplot (jittered) of the linear relationship of the ASD and the PCOP. Notes: ASD = Aging Semantic Differential scale, PCOP = Perspectives on Caring for Older People scale

The linear relationship between the PCOP sum score and the ASD sum score is shown in Fig. 1. Pearson correlation showed a moderate negative correlation (r = -0.303; *p* ≤ 0.001), indicating a relationship between the PCOP and the ASD.

### Multiple linear regression models regarding the PCOP and the ASD

Two multiple linear regression models with the PCOP and ASD scores as dependent variables were conducted. The application of both models yielded statistically significant results (*p* ≤ 0.001). The following six factors had a statistically significant positive association with nurses’ attitudes towards the care of adults aged 80 years and older (PCOP as a dependent variable): (1) being from Austria, (2) having worked in long-term care for a longer period, (3) having a clinical work focus, (4) perceiving contact with care receivers aged 80 years and older as positive (PCS), (5) not perceiving contact with care receivers as negative (NCS), and (6) knowing the meaning of ageism. The following four factors had a statistically significant positive association with nurses’ general attitudes (ASD as dependent variable): (1) perceiving contact with care receivers as positive (PCS), (2) having a lower score regarding negative contact (NCS), (3) having a close personal relationship with an older adult (being able to discuss personal topics often/always), and (4) not thinking of a gender while answering the ASD compared to thinking of a male person. Tables 4 and 5 show the regression coefficients for all variables included in the multiple linear regression models.

**Table 4:** Multiple linear regression models with the PCOP sum score as a dependent variable

	**Independent variable**	**Category 1 (reference category 0)**	***B***	**95*% CI* for *B***		***p-value***
				***LB***	***HB***	
**Dependent variable = PCOP sum score**	Origin	Non-Austrian (Austrian)	-1.080	-1.899	-0.261	**0.010**
	Years working in long-term care	Continuous variable	0.053	0.025	0.080	**≤ 0.001**
	Clinical focus	No (yes)	-0.723	-1.257	-0.188	**0.008**
	PCS multiplied score	Continuous variable	0.187	0.155	0.219	**≤ 0.001**
	NCS multiplied score	Continuous variable	-0.134	-0.192	-0.076	**≤ 0.001**
	Interaction with person in need of care ≥ 80	Often (Occasionally)	0.199	-0.444	0.842	0.544
	Able to discuss personal topics	Few/no (Many/all)	-0.135	-0.670	0.400	0.620
	Knowing the meaning of ageism	No (yes)	-0.597	-1.184	-0.011	**0.046**
	Knowing the meaning of ageism (no)	Unsure (yes)	0.091	-0.559	0.741	0.784

*B* unstandardised regression coefficient, *CI* confidence interval, *LB* lower bound, *HB* higher bound, *PCS* Positive Contact Scale, *NCS* Negative Contact Scale, *PCOP* Perspectives on Caring for Older People scale, *p*- values ≤ 0.05 in bold

*n* = 619; *adjusted R*^*2* ^= 0.324; *p* = ≤ 0.001

**Table 5:** Multiple linear regression models with the ASD sum score as a dependent variable

	**Independent variable**	**Category 1 (reference category 0)**	***B***	**95*% CI* for *B***		***p-value***
				***LB***	***HB***	
Dependent variable = ASD sum score	Origin (non-Austrian)	Non-Austrian (Austrian)	3.594	-1.328	8.515	0.152
	Years working in long-term care	Continuous variable	-0.052	-0.221	0.116	0.543
	PCS multiplied score	Continuous variable	-0.650	-0.844	-0.457	**≤ 0.001**
	NCS multiplied score	Continuous variable	1.051	0.694	1.407	**≤ 0.001**
	Interaction with person in need of care ≥ 80	Often (Occasionally)	-0.438	-4.428	3.552	0.829
	Able to discuss personal topics	Few/no (Many/all)	3.743	0.437	7.050	**0.027**
	Thinking of a gender while answering the ASD	Female (No)	-4.216	-8.464	0.033	0.052
	Thinking of a gender while answering the ASD	Male (No)	-12.115	-22.336	-1.895	**0.020**
	Thinking of a gender while answering the ASD	Other (No)	1.243	-10.703	13.188	0.838

*B* unstandardised regression coefficient, *CI* confidence interval, *LB* lower bound, *HB* higher bound, *PCS* Positive Contact Scale, *NCS* Negative Contact Scale, *ASD* Aging Semantic Differential scale, *p*- values ≤ 0.05 in bold

*n* = 648; *adjusted R*^*2* ^= 0.194; *p* = ≤ 0.001

## Discussion

In this study, three aims were investigated. The first aim was to assess Austrian nurses’ attitudes towards adults aged 80 years and older in general and towards their care in long-term care settings. The second aim was to compare nurses’ attitudes in the residential care and home care settings. The third aim was to investigate which factors influence nurses’ attitudes, such as the quality of contact with care receivers aged 80 years and older.

Compared to nurses working in home care, nurses working in long-term care stated statistically significantly more often that they perceived their contact with care receivers as positive. Attitudes towards adults aged 80 years and older were, in general, neutral to positive, and statistically significantly more positive attitudes were expressed by nurses working in home care. Caring for care receivers aged 80 years and older was perceived as positive, and no difference in terms of settings was observed. Perceiving contact with care receivers as positive was associated with a more positive attitude towards adults aged 80 and older in general and towards their care. Furthermore, having a good personal relationship with an older family member or friend was positively associated with nurses’ general attitudes. Having worked more years in long-term care was also positively associated with positive attitudes towards their care. The setting had no influence on the general attitudes or the perception regarding the care of adults aged 80 and older.

According to Allport’s 1950 [20] contact hypothesis, we hypothesised that the quality of contact with older care receivers influences nurses’ attitudes towards them and their care. The study results show that having a good relationship with an older adult as well as having had positive contact experiences with an older care receiver influenced nurses’ attitudes towards older adults significantly. Nurses’ perception of care is also influenced by a positive contact experience and by having worked longer in long-term care. This finding is in line with Allport’s 1950 [20] contact hypothesis: Nurses who have positive and meaningful contact with older adults in their personal lives or with care receivers at work life can improve their attitudes towards older adults, and this, in turn, can diminish prejudice. A study by Cadieux et al. [25] drew similar conclusions with respect to younger adults’ attitudes. Younger adults who had more contact with older adults were less likely to stereotype older adults as incompetent, for example.

Because nurses are in regular and close contact with frail and care-dependent older adults, they may have biased attitudes; namely, they might develop more negative attitudes towards older adults than other members of the public [14]. Therefore, Cadieux’s et al. [25] findings from the general population might not be comparable to our findings for nurses working in health care settings. Nurses may perceive their contact experiences differently because they frequently work with frail or care-dependent older adults [15], and this even more so if difficulties in providing care occur [26]. In addition, their contact might be affected by difficult and stressful working conditions such as a lack of time or resources [46]. The results in Drury et al. [18], using Allport’s contact hypothesis, also confirm the influence of nurses’ contact with older care receivers on their attitudes or on ageism. Their results suggest that experiencing positive contact had more influence on undisguised forms of ageism and that negative contact had a stronger influence on subtle forms of ageism. A study by Kusumastuti et al. [47] also used Allport’s contact hypothesis when examining medical students’ attitudes towards older adults before and after clinical placement and concluded similarly that the quality of contact seems to be crucial to forming attitudes.

In this study, we only examined ageist attitudes in the form of explicit, other-directed ageism with regard to stereotypes or prejudice [48, 49] by means of the ASD. According to this scale, negative contact experiences with an older care receiver had a stronger influence on nurses’ negative attitudes towards adults aged 80 years and older in general. Uğurlu et al. [26] reported similar results when they discovered that experienced difficulties in geriatric care negatively influenced nurses’ tendencies to display ageism. They also found that experiencing positive contact led nurses to more strongly perceive geriatric care as positive. These results suggest that experiencing positive contact with an older care receiver might improve nurses’ or nursing students’ willingness to work in geriatric care. A study by Jang et al. [50] investigated whether the quality of contact between nursing students and older care receivers positively influenced nursing students’ willingness to work with older adults, but no support for this hypothesis was found. Rathnayake et al. [51] discovered that intergenerational contact had a positive influence on nursing students’ attitudes towards older people and, in turn, a positive influence on their willingness to work with older adults. A meta-analysis showed that intergenerational contact in combination with educational measures has a significant effect on people’s attitude towards older adults [52]. To encourage intergenerational contact, the WHO [53] has developed a guide to design intergenerational activities and education programmes. These activities should encourage bonding between representatives of different generations and address topics that are of importance to members of different generations. These could include knowledge about ageing, age-friendly communities, gardening, or arts and crafts [53–55]. Nursing and care-relevant topics, such as wishes for delivering or receiving care or ageism in health care, could also be considered. This is also supported by the Positive Education about Aging and Contact Experiences (PEACE) model by Levy [56]. This model includes two approaches to improving attitudes towards older adults. (1) Education including facts on ageing and positive role models, and (2) positive contact experiences. Furthermore, the University of Hong Kong, China, developed an intergenerational participatory co-design project to address the issue of students’ negative attitudes toward older adults [57]. The Optimal Quality Intergenerational Interaction Model was used to guide the project. This framework was developed to inform intergenerational contact activities and programmes in China [58]. The co-design approach significantly changed students’ attitudes towards older adults [57]. This approach might also be considered for nurses in long-term care.

Some evidence suggests the existence of age stereotypes among nurses [29, 59]. Nevertheless, the research on nurses’ attitudes towards older adults in long-term care is limited. One study found that assistant nurses displayed positive attitudes towards older adults [60]; findings which are in line with the results of this study. In general, the results reported for nurses’ attitudes towards older adults range from positive to negative attitudes [15]. A previous study by Lampersberger et al. [19] reported assessment results for nurses’ attitudes towards adults aged 80 years and older in several settings, although mainly acute care nurses participated in the study. The findings were similar to those of the current study, with nurses displaying neutral attitudes towards older adults in general and reporting positive perceptions of caring for older adults [19].

### Limitations

Although many participants took part in this study, nursing managers and nurses interested in gerontology and the care of older adults might have been more motivated to forward or accept the invitation to participate in this study due to the convenience sampling method used. Thus, the attitudes reported might be more positive. By using a non-representative convenience sampling strategy, we may have introduced this selection bias in our study, which has to be considered when interpreting the study results. While a random sampling method would have reduced the risk of selection bias, we were not able to obtain a full list of possible participants and, consequently, unable to implement this strategy [31]. This study design does not allow us to draw conclusions about causality, and only influencing factors can be identified by assessing these results [31]. Due to the lack of an imputation method for missing data, a number of cases had to be deleted in the multiple linear regression models and when analysing the scales. This might have affected the strength of the models.

### Implications

The study findings show that nurses working in long-term care hold neutral to positive attitudes towards adults aged 80 years and older and their care. To facilitate further changes in their attitudes, we recommend encouraging positively perceived contact. One way to increase the frequency of positively perceived contact might be to implement intergenerational education programmes or activities by using the WHO [53] guide or a co-design project using the Optimal Quality Intergenerational Interaction Model. An activity or educational topic which is of interest for both age groups should be chosen. In nursing care, this could be knowledge about ageing [54, 55] or courses on ageism. We recommend conducting further research on the effectiveness of the co-design approach in the long-term care setting and the use of the Optimal Quality Intergenerational Interaction Model. With regard to educational interventions for long-term care nurses, these study results can be used to raise awareness of ageism in health care. To obtain more insight into ageism in nursing care, we recommend conducting further investigations using quantitative methods, such as cross-sectional studies that include ageism scales, or qualitative study methods, such as observations. We recommend using a random sample instead of a convenience sample to increase representativity and, consequently, obtain more powerful results. Subsequently, a comparison between settings can be made to tailor interventions to different health care settings. To obtain a broader perspective of attitudes towards older adults and ageism, researchers should investigate how older adults experience interactions with nurses in health care, using qualitative study methods such as interviews. Studies that include randomised controlled trials to test nursing-specific intergenerational activities or education programmes in long-term care need to be carried out to be able to develop an effective programme for nurses and older adults as care receivers. Although the PNCS and PCOP were tested with regard to internal reliability, further psychometric testing in terms of a factor analysis is recommended; this would enable the German versions of these scales to be used in the long-term care setting.

## Conclusion

One encouraging result of this study is that nurses working in long-term care hold neutral to positive attitudes towards older adults and view their care as positive. We will face more challenges caring for older adults as people live longer, and we should make an effort to create a care and work environment that is perceived positively by both nurses and older adults. Implementing intergenerational activities and education programmes might contribute to delivering sustainable high-quality and integrated care. To implement intergenerational activities, the PEACE model or the WHO guide to design intergenerational activities and education programmes can be used. Furthermore, these study results can be used for educational interventions in long-term care to inform nurses on current attitudes of nurses towards older adults in Austria to raise awareness of ageism. This intergenerational contact may prevent or counter ageism and, depending on the chosen topic, can help to shape the care landscape.

## Supplementary Information

Supplementary Material 1.

## Data Availability

The dataset used in the current study is available from the corresponding author on reasonable request.

## Abbreviations

PNCS: Positive and Negative Contact Scales
STROBE: Strengthening the Reporting of Observational Studies in Epidemiology
ASD: Aging Semantic Differential scale
PCOP: Perspectives on Caring for Older People scale
PCS: Positive Contact Scale
NCS: Negative Contact Scale
VIF: Variance inflation factors
PEACE: Positive Education about Aging and Contact Experiences
