# Supplementary material for: Nurses’ perspectives on old age and caring for adults aged 80 years and older: a cross-sectional study in long-term care
Source: BMC Nurs. 2024 Nov 20;23:850. doi: 10.1186/s12912-024-02503-w (PMC11580359; doi:10.1186/s12912-024-02503-w)
Supplement: Supplementary file 1 — Supplementary Material 1. [file 12912_2024_2503_MOESM1_ESM.pdf]

Additional File 1

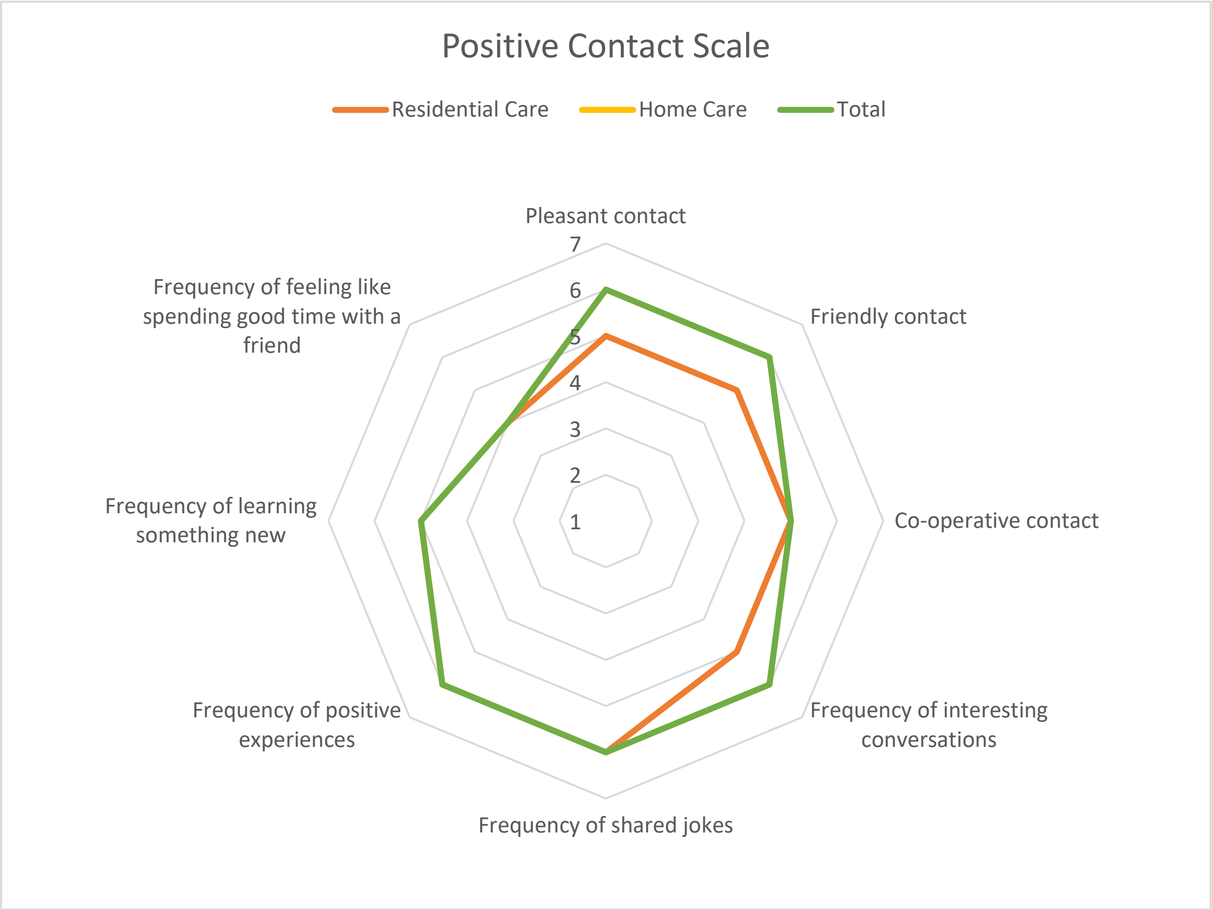

1 = None/never
2
3
4
5
6
7 = All/very often

Figure 1: *Radar charts for the item means of the Positive Contact Scale (PCS), for residential care, home care, and total*

Note: Item means overlap

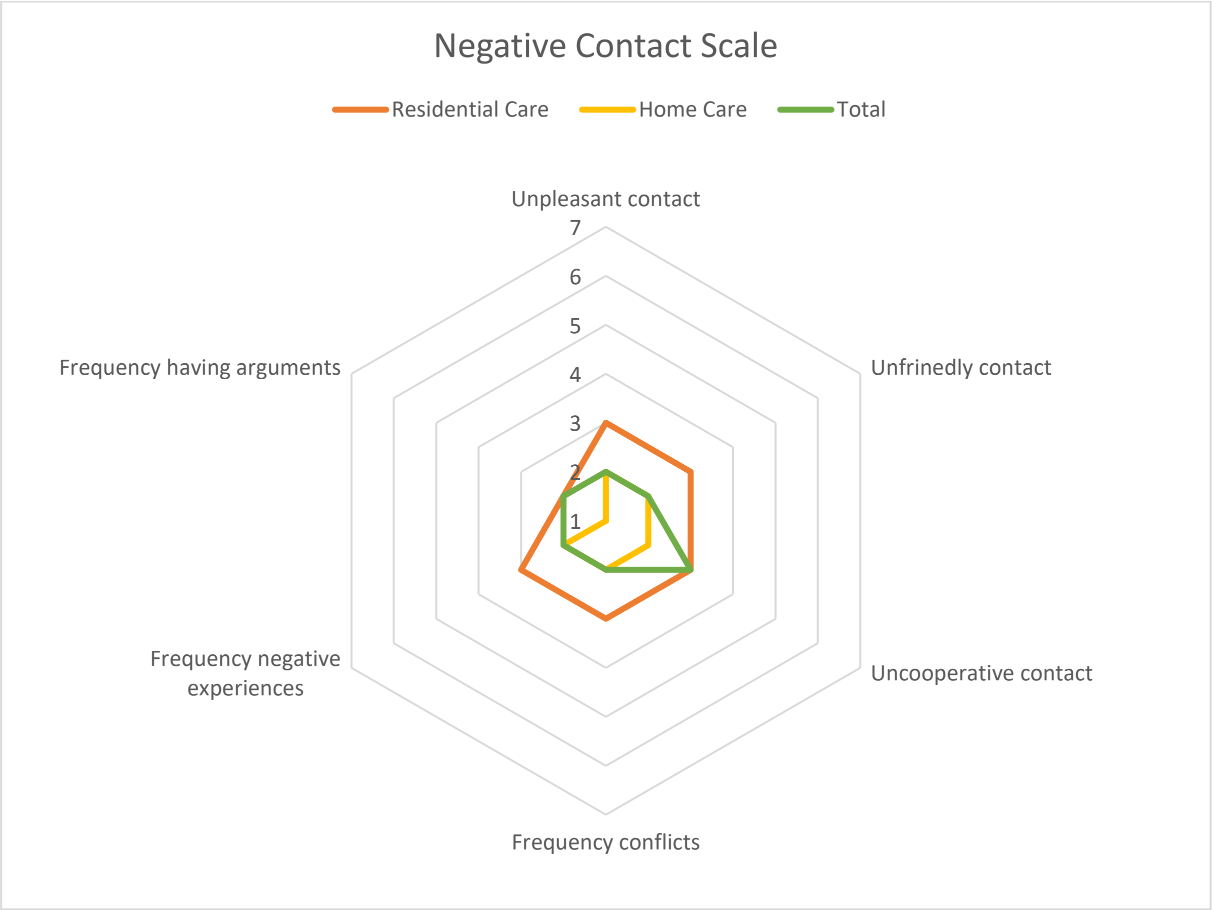

1 = None/never
2
3
4
5
6
7 = All/very often

Figure 2: *Radar charts for the item means of the Negative Contact Scale (NCS), for residential care, home care, and total*

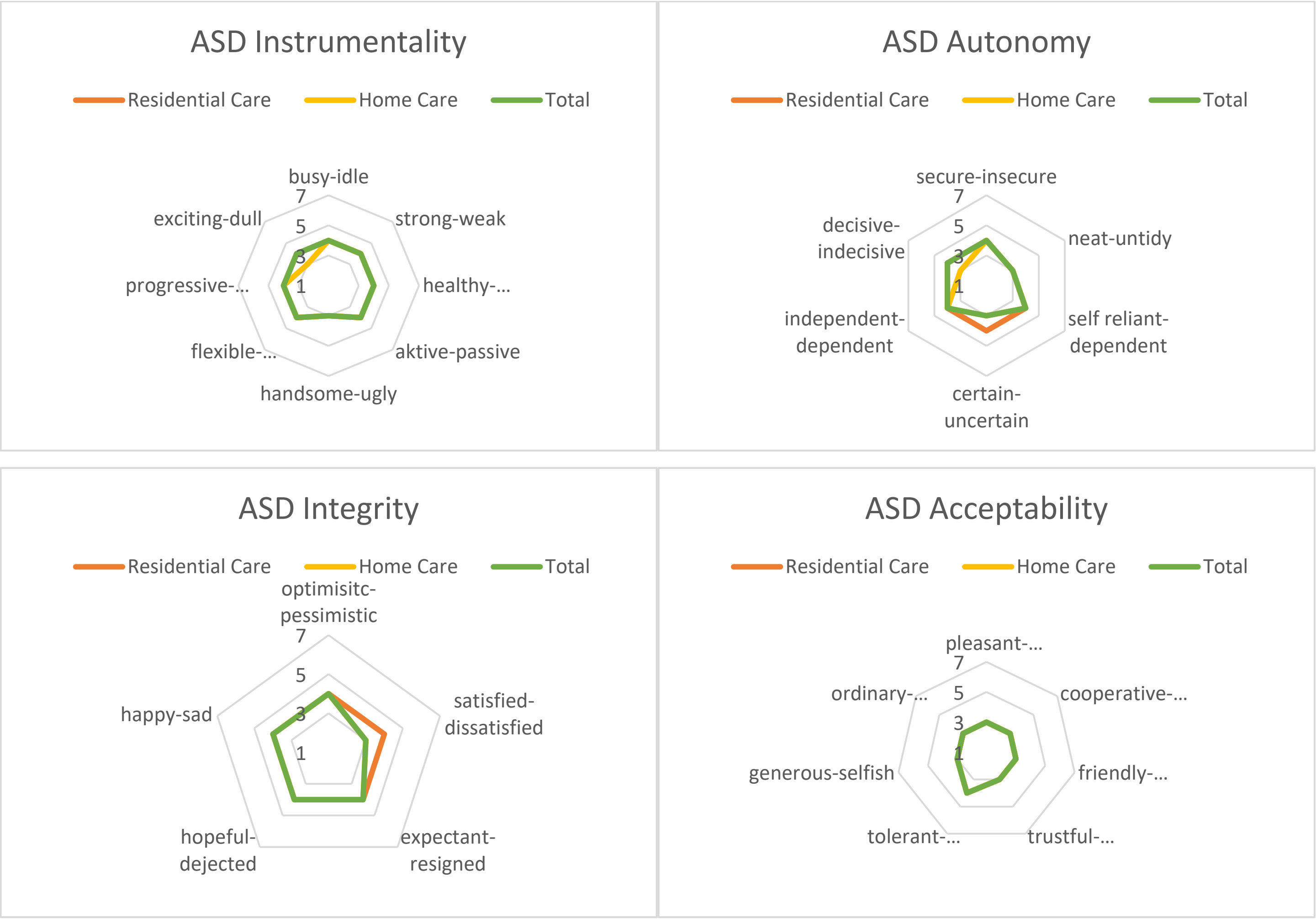

1 = Very positive
2 = Positive
3 = Rather positive
4 = Neutral
5 = Rather negative
6 = Negative
7 = Very negative

Figure 3: *Radar charts for the item means of the Ageing Semantic Differential (ASD) for residential care, home care, and total*

Note: Item means overlap

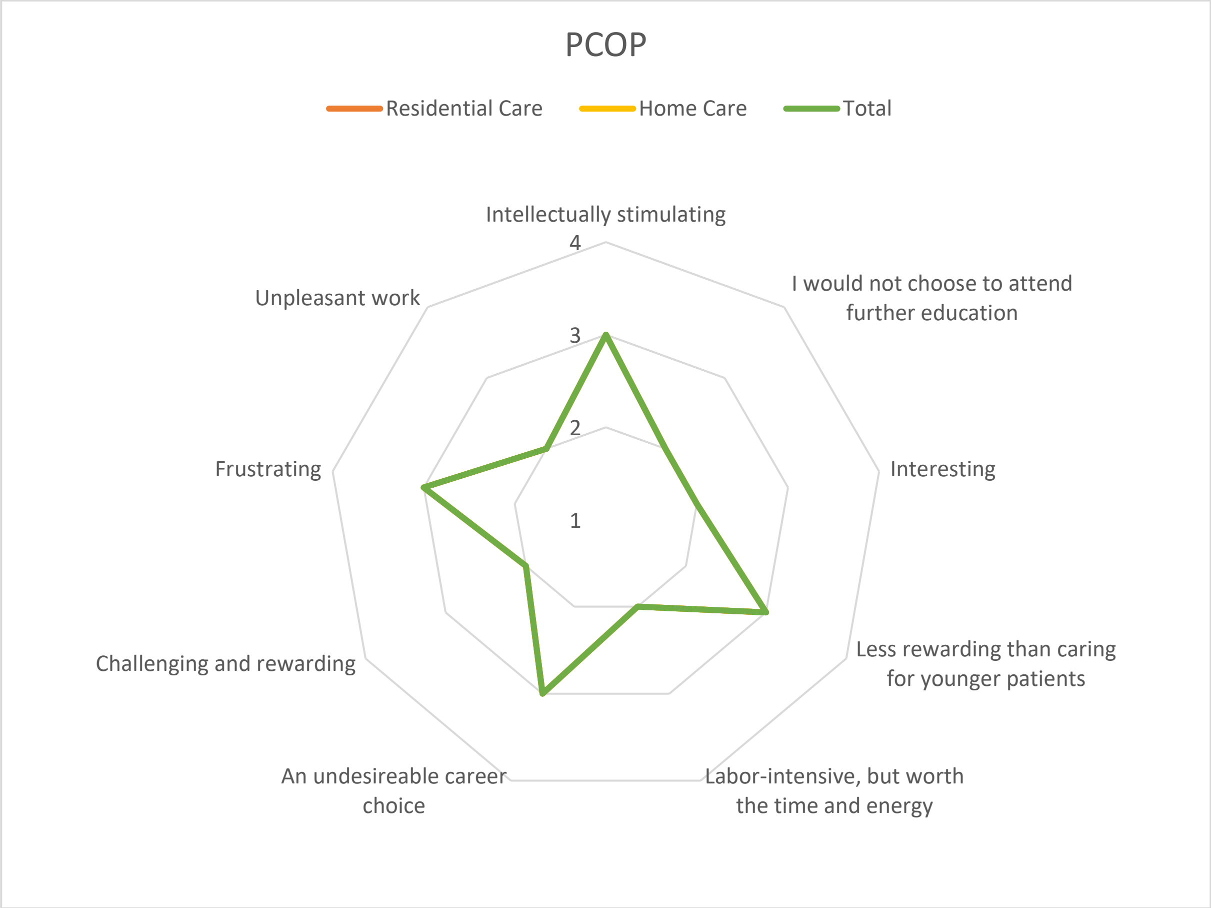

1 = Strongly disagree
2 = Disagree
3 = Agree
4 = Strongly agree

Figure 4: *Radar charts for the item means of the on Caring for Older People scale (PCOP) for residential care, home care, and total*

Note: Item means overlap
